# Nectin-3 modulates the structural plasticity of dentate granule cells and long-term memory

**DOI:** 10.1038/tp.2017.196

**Published:** 2017-09-05

**Authors:** X-X Wang, J-T Li, X-M Xie, Y Gu, T-M Si, M V Schmidt, X-D Wang

**Affiliations:** 1 Department of Neurobiology, Key Laboratory of Medical Neurobiology of Ministry of Health of China, Zhejiang Province Key Laboratory of Neurobiology, Zhejiang University School of Medicine, Hangzhou, China; 2 National Clinical Research Center for Mental Disorders (Peking University Sixth Hospital/Institute of Mental Health), Key Laboratory of Mental Health, Ministry of Health (Peking University), Beijing, China; 3 Li Dak Sum and Yip Yio Chin Center for Stem Cells and Regenerative Medicine, Key Laboratory of Tissue Engineering and Regenerative Medicine of Zhejiang Province, Zhejiang University School of Medicine, Hangzhou, China; 4 Department of Stress Neurobiology and Neurogenetics, Max Planck Institute of Psychiatry, Munich, Germany

## Abstract

Nectin-3, a cell adhesion molecule enriched in hippocampal neurons, has been implicated in stress-related cognitive disorders. Nectin-3 is expressed by granule cells in the dentate gyrus (DG), but it remains unclear whether nectin-3 in DG modulates the structural plasticity of dentate granule cells and hippocampus-dependent memory. In this study, we found that DG nectin-3 expression levels were developmentally regulated and reduced by early postnatal stress exposure in adult mice. Most importantly, knockdown of nectin-3 levels in all DG neuron populations by adeno-associated virus (AAV) mimicked the cognitive effects of early-life stress, and impaired long-term spatial memory and temporal order memory. Moreover, AAV-mediated DG nectin-3 knockdown increased the density of doublecortin-immunoreactive differentiating cells under proliferation and calretinin-immunoreactive immature neurons, but markedly decreased calbindin immunoreactivity, indicating that nectin-3 modulates the differentiation and maturation of adult-born DG granule cells. Using retrovirus to target newly generated DG neurons, we found that selective nectin-3 knockdown in new DG neurons also impaired long-term spatial memory. In addition, suppressing nectin-3 expression in new DG neurons evoked a reduction of dendritic spines, especially thin spines. Our data indicate that nectin-3 expressed in DG neurons may modulate adult neurogenesis, dendritic spine plasticity and the cognitive effects of early-life stress.

## Introduction

Repeated exposure to adverse life events markedly increases the risk for major psychiatric disorders.^1, 2, 3^ Severely stressful experiences during the postnatal stage may suppress the developmental trajectory of hippocampal principle neurons,^4^ and induce persistent abnormalities in hippocampal plasticity and function.^5, 6, 7^ The dentate gyrus (DG) serves a critical role in cognitive and emotional information processing.^8, 9^ During the early postnatal period, DG cells undergo orchestrated developmental events ranging from the proliferation and migration of new neurons to synaptic formation and circuit integration.^10, 11^ Exposure to stressors during this critical time window disrupts developmental and adult neurogenesis as well as synaptic plasticity in DG neurons, and impairs cognitive performance later in life.^12, 13, 14, 15^

A deeper understanding of the molecular substrates underlying the reprogramming effects of early-life stress on DG neurons will provide insight into the regulatory mechanisms of DG development and the pathophysiology of stress-related disorders. The roles of stress mediators, especially glucocorticoids and corticotropin-releasing hormone (CRH), as well as nutritional and immune factors in the negative consequences of early-life stress on DG structure and function have been unraveled,^6, 10, 16, 17^ which are also dependent on genetic background to exert effects.^18^ Recent evidence suggests that augmented hippocampal CRH–CRH receptor 1 (CRHR1) signaling mediates the enduring effects of early postnatal stress, leading to dendritic simplification, spine elimination, synaptic impairment and cognitive deficits.^4, 19, 20^ Notably, early-life stress reduces the expression levels of nectin-3, a cell adhesion molecule primarily localized at adherens junctions, in the neonatal and adult hippocampus via CRH–CRHR1 signaling.^21, 22, 23^ Moreover, nectin-3 knockdown in the CA3 region mimics the effects of early-life stress on spine remodeling and cognitive performance, whereas nectin-3 overexpression in CA3 attenuates stress-evoked spine loss and cognitive deficits.^21^ These findings highlight nectin-3 as an important molecule associated with early-life stress and a promising target to treat stress-related cognitive disorders.

So far, the effects of stress on nectin-3 expression in the CA3 or CA1 region have been investigated.^21, 24, 25^ However, it remains unclear whether stress dysregulates nectin-3 levels in DG neurons and whether impaired nectin-3-mediated cell adhesion contributes to the stress effects on DG structure and function. In this study, we used adeno-associated virus (AAV) to suppress nectin-3 protein expression in both immature and mature DG neurons, and assessed the influences of DG nectin-3 knockdown on anxiety-related behavior and memory. Subsequently, we used retrovirus (RV) to specifically target nectin-3 expressed in newly generated dentate granule cells, and examined its impact on dendritic spine density and behavior. Our findings indicate that nectin-3 knockdown in either old or young DG neurons may compromise hippocampus-dependent spatial memory, which may further contribute to the adverse effects of early-life stress.

## Materials and methods

### Animals and housing

Adult male and female C57BL/6N mice (12 weeks old; Vital River Laboratories, Beijing, China) were used for breeding in Experiment 1. Each female was housed with one male for 2 weeks and then single-housed. Pregnant females were monitored daily, and the day of delivery was defined as postnatal day 0. Only male offspring were used. In Experiments 2 and 3, adult male mice (8 weeks old) were single-housed after habituation. All animals were held under standard conditions (12:12 h light/dark cycle, lights on at 0800 hours, temperature 22±2 °C) with free access to food and water, and were randomly assigned to each group. The experiments were approved by the Animal Advisory Committee at Zhejiang University and were performed in compliance with the National Institute of Health’s Guide for the Use and Care of Laboratory Animals.

### Experimental design

Experiment 1 examined the effects of early-life stress on nectin-3 protein expression and spine density in adult DG neurons. Mice were killed at 12 weeks of age. Experiment 2 used AAV to assess the effects of nectin-3 knockdown in all DG neuron populations on adult neurogenesis, anxiety-related behavior and hippocampus-dependent memory. Experiment 3 used RV to specifically target adult-born granule cells and evaluated the effects of nectin-3 knockdown in young DG neurons on dendritic spine plasticity and behavior. In Experiments 2 and 3, mice (12 weeks old) were killed 24 h after behavioral testing, and their brains were processed for further analysis. Mice used to validate the knockdown efficiency of RV-short hairpin RNA against nectin-3 (shNEC) were killed at 8 weeks of age.

### Early-life stress procedure

The limited nesting and bedding material paradigm was performed as detailed previously.^4, 26^ On the morning of P2, control dams were provided with a sufficient amount of nesting material (4.8 g of Nestlets, Indulab, Gams, Switzerland) and 500 ml of sawdust bedding. In the ‘stress’ cages, the dams were provided with a limited quantity of nesting material (1.2 g of Nestlets), which was placed on an aluminum mesh platform (McNichols, Tampa, FL, USA). The stress procedure ended on the morning of P9. Male mice were weaned on P28 and group-housed in three to four per cage.

### Virus-mediated *in vivo* nectin-3 knockdown

We used AAV2/8 and RV vectors (Obio Technology, Shanghai, China) to suppress nectin-3 expression in DG neurons. The short hairpin RNA (shRNA) sequence (5′-TGTGTCCTGGAGGCGGCAAAGCACAACTT-3′) targeting nectin-3 has been validated previously.^21^ AAV-shSCR (AAV2/8-U6-scrambled.shRNA-terminator-CAG-EGFP-WPRE-BGH-polyA, 3.5 × 10^12^ viral genomes per ml), AAV-shNEC (AAV2/8-U6-Nectin-3.shRNA-terminator-CAG-EGFP-WPRE-BGH-polyA, 3.9 × 10^12^ viral genomes per ml), RV-shSCR (RV-EF1A-EGFP-U6-scrambled.shRNA-terminator-CAG-WPRE-BGH-polyA, 1.8 × 10^10^ viral genomes per ml) and RV-shNEC (RV-EF1A-EGFP-U6-Nectin-3.shRNA-terminator-CAG-WPRE-BGH-polyA, 4.2 × 10^10^ viral genomes per ml) were generated and purified by Obio Technology.

Stereotaxic surgery and microinjection were performed as previously described.^27^ Briefly, 0.5 μl of AAV or 0.75 μl of RV was delivered bilaterally to the dorsal DG (1.8 mm posterior to bregma, 1.2 mm lateral from midline and 1.65 mm dorsoventral from dura) of 8-week-old adult mice over a 15-min period. The micropipette was left in the site for additional 5 min. Mice were given a 4-week recovery period before behavioral testing to allow sufficient viral infection.

To validate the knockdown efficiency of RV-shNEC, 0.1 μl of RV-shSCR or RV-shNEC was delivered bilaterally to the dorsal hippocampus of male pups (1 mm anterior to lambda, 1.2 mm lateral from midline and 1.25 mm dorsoventral from dura) on P2, when hippocampal neurogenesis is more active than adulthood. Mice were killed at 8 weeks of age.

### Behavioral testing

Behavioral tests were performed between 0900 and 1600 hours and scored by ANY-maze 4.98 (Stoelting, Wood Dale, IL, USA) as detailed previously.^21, 28, 29^

#### Open field test

Mice were placed in the open field arena (50 × 50 × 50 cm^3^) made of gray polyvinyl chloride and were evenly illuminated at 10 lux. The time in the center zone (25 × 25 cm^2^) and total distance traveled were analyzed for 10 min.

#### Light–dark box test

Mice were placed in the dark chamber (15 × 20 × 25 cm^3^, 10  lux), facing the brightly illuminated chamber (30 × 20 × 25 cm^3^, 650 lux). The two chambers were connected by a 4-cm-long tunnel. During the 5-min test, the time spent in the light chamber was measured.

#### Elevated plus maze test

The elevated plus maze was made of gray polyvinyl chloride with two opposing open arms (30 × 5 × 0.5 cm^3^, 40 lux) and two opposing enclosed arms (30 × 5 × 15 cm^3^, 10 lux) connected by a central platform (5 × 5 cm^2^). Mice were placed in the center of the maze and allowed to explore for 5 min. The time spent in the open arms was recorded.

#### Y-maze test

The Y-maze apparatus was made of gray polyvinyl chloride with three symmetrical arms (30 × 10 × 15 cm^3^) and illuminated at 10 lux. Prominent extra-maze spatial cues were provided. Mice were placed in the end of one arm and allowed to freely explore for 5 min. Three consecutive choices of all three arms were counted as an alternation. The percentage of spontaneous alternation was determined by dividing the total number of alternations by the total number of choices minus 2.

#### Spatial object recognition test

The test was performed in the open field arena under low illumination (10 lux). Prominent spatial cues were provided. On 1 day before testing, the mice were habituated to the testing environment for 10 min on two trials separated by an intertrial interval of 60 min. The testing procedure consisted of two sessions separated by an intertrial interval of 60 min. In the sample phase, mice were presented with two identical circular cones and allowed to explore for 10 min. During the 10-min retrieval trial, mice were presented with a non-displaced (known) object and a relocated (novel) one, and the time spent exploring each object was measured. The discrimination index (DI) was calculated as follows: DI=100% × (time with the novel object−time with the known object)/time with both objects.

#### Temporal order memory test

The test was performed in the open field arena under low illumination (10 lux). No spatial cue was provided. On 1 day before testing, mice were habituated to the testing environment for 10 min on three consecutive trails separated by 60 min of intertrial intervals. The testing procedure consisted of three sessions separated by 60 min of intertrial intervals. In sample phases 1 and 2 (10 min each), two cubes and two cylinders were presented, respectively. In the retrieval phase (10 min), one cube (the ‘remote’ object) and one cylinder (the ‘recent’ object) were presented. DI was calculated as: 100% × (time with the remote object−time with the recent object)/time with both objects.

### Immunostaining and image analysis

Mice were anesthetized with sodium pentobarbital (200 mg kg^−1^) and transcardially perfused with 0.9% saline followed by 4% buffered paraformaldehyde. Following post fixation and cryoprotection, serial coronal sections were prepared through the dorsal hippocampus (Bregma −1.22 to −2.92 mm) at 30 μm thickness and 300 μm intervals using a cryostat (Leica, Wetzlar, Germany). The following primary antibodies were used for immunohistochemistry and immunofluorescence: rabbit anti-nectin-3 (1:1000; ab63931, Abcam, Cambridge, UK), rabbit anti-minichromosome maintenance complex component 2 (MCM2; 1:1000; 4007, Cell Signaling, Danvers, MA, USA), rabbit anti-doublecortin (1:1000; 4604, Cell Signaling), rabbit anti-calretinin (1:5000; 7697, Swant, Marly, Switzerland), rabbit anti-calbindin D-28k (referred to as calbindin hereafter; 1:20 000; CB-38, Swant) and rabbit anti-green fluorescent protein (GFP; 1:5000; sc-8334, Santa Cruz Biotechnology, Santa Cruz, CA, USA).

For immunohistochemistry, free-floating sections were simultaneously treated with 3% hydrogen peroxide (10 min) followed by 1% normal goat serum (1 h), and were then labeled with primary antibodies overnight at 4 °C. The next day, sections were rinsed and incubated with a biotinylated goat anti-rabbit secondary antibody (Zhongshan Golden Bridge Biotechnology, Beijing, China) for 2 h at room temperature. After rinsing, the 3,3′-Diaminobenzidine Horseradish Peroxidase Color Development Kit (Zhongshan Golden Bridge) was used for staining. Finally, the sections were transferred onto slides and coverslipped.

For immunofluorescence, the sections were treated with 1% normal donkey serum for 1 h and labeled with the rabbit anti-GFP antibody overnight at 4 °C. The next day, the sections were rinsed and labeled with Alexa Fluor 488-conjugated donkey anti-rabbit secondary antibody (1:500; Invitrogen, Carlsbad, CA, USA) for 3 h at room temperature. After rinsing, the sections were transferred onto slides and coverslipped with Vectashield containing 4′,6-diamidino-2-phenylindole (Vector Laboratories, Burlingame, CA, USA).

To quantify the immunoreactivity of nectin-3 and calbindin, images from six sections per animal were acquired at × 100 using the Olympus BX61 microscope and analyzed by ImageJ (National Institute of Health, Bethesda, MD, USA) as described previously.^22^ Relative protein levels were determined by the differences in optical density values between the region of interest (Supplementary Figure S1A) and corpus callosum from the same section, which generally lacks staining and was considered as the background. Results were normalized by taking the mean value of the control group as 100%.

To quantify the density of MCM2-, doublecortin- and calretinin-immunoreactive cells in the subgranular zone of dorsal DG, cells were counted at × 400 using an Olympus BX61 microscope (Olympus, Tokyo, Japan) by an investigator blind to the experimental conditions. Doublecortin-positive cells were further classified into six categories (Supplementary Figure S1B) based on dendritic morphology as described previously.^30^ For each animal, both hemispheres on six sections (300 μm apart) were analyzed. The cell counts were multiplied by 10 (series number) to estimate the total number of immunoreactive cells in the dorsal DG. To estimate the volume of the granule cell layer (GCL) of dorsal DG, images were captured at × 100 using a DP72 camera (Olympus) fitted to the microscope. The GCL was outlined and its area was measured using the ImageJ Software. The area of GCL was multiplied by 10 and then by the thickness of the sections (30 μm) to calculate dorsal DG GCL volume. Cell counts were presented as the number of immunoreactive cells per mm.^3^

To analyze the dendritic spines of RV-infected young DG granule cells, images (800 × 800 pixel^2^) covering the medial molecular layer (ML) of dorsal DG were obtained with an Olympus IX81-FV1000 laser-scanning confocal microscope (Olympus). Dendritic segments (30–70 μm in length, eight dendrites per mouse sampled from both suprapyramidal and infrapyramidal blades) were scanned at 0.1-μm intervals along the *z* axis using a × 60 oil-immersion objective (numerical aperture 1.35) with a × 2.6 digital zoom, yielding a voxel size of 0.1 × 0.1 × 0.1 μm^3^. The z stack images were deconvolved using the AutoQuant X3 software (Media Cybernetics, Silver Spring, MD, USA) and analyzed with the NeuronStudio software (http://research.mssm.edu/cnic/tools-ns.html) by an investigator blind to the experimental conditions. Dendritic spines were categorized as thin, mushroom and stubby subtypes based on established criteria.^28, 31^ Spine density was expressed as the number of spines per 10 μm of dendrite.

### Golgi–Cox staining and the analysis of dendritic spines

Brains were immersed in the Golgi–Cox solution for 14 days and transferred to the 30% sucrose solution for 2–5 days in the dark at room temperature. Coronal sections (120 μm) were prepared using a vibratome (Microm HM 650V, Thermo Scientific, Walldorf, Germany) and processed as described previously.^28^ Bright-field z-series images of fully impregnated DG granule cells were acquired at × 600 using the DP72 camera fitted to the Olympus BX61 microscope equipped with a ProScan motorized stage (Prior Scientific, Rockland, MA, USA; z-step=1 μm). Dendritic segments (40–70 μm in length, four from the suprapyramidal blade and four from the infrapyramidal blade for each mouse) in the medial ML of DG were reconstructed and dendritic spines were analyzed using NeuronStudio as described above.

### Western blot

Western blot was performed as described previously.^22^ Hippocampi from both hemispheres were dissected. For Experiment 2, DG and CA1–3 regions were further separated according to an established protocol.^32^ Hippocampal tissues were homogenized in ice-cold lysis buffer and centrifuged at 10 000 r.p.m. for 20 min at 4 °C. Protein concentrations were determined using a bicinchoninic acid protein assay kit (Pierce, Rockford, IL, USA). Samples containing 20 μg of protein were resolved by 10% sodium dodecyl sulfate-polyacrylamide gels, and transferred onto polyvinylidene difluoride membranes (Millipore, Bedford, MA, USA). Membranes were labeled with rabbit anti-nectin-3 (1:2000; Abcam), mouse anti-actin (1:10 000; E021020-01, EarthOx, Millbrae, CA, USA) or mouse anti-GFP (1:20 000; sc-9996, Santa Cruz) overnight at 4 °C. Following incubation with horseradish peroxidase-conjugated secondary antibodies (1:2000, Sigma-Aldrich, St Louis, MO, USA) for 3 h at room temperature, bands were visualized using an enhanced chemiluminescence system (Pierce) and quantified with densitometry (Quantity One 4.2, Bio-Rad, Hercules, CA, USA). All results were normalized by taking the value of the control group as 100%. Each assay was repeated for at least three times.

### Statistical analysis

SPSS 16.0 (SPSS, Chicago, IL, USA) was used to perform statistical analyses. Nectin-3 protein levels across developmental stages were analyzed by one-way analysis of variance (ANOVA) followed by Tukey’s *post hoc* test when appropriate. For the analysis of time exploring the objects during the acquisition phase(s) of the spatial object recognition and temporal order memory tasks, two-way ANOVA and two-way repeated measures ANOVA were performed, respectively. The two-tailed Student’s *t*-test was used to compare pairs of means. For dendritic spine analysis, statistics were performed by averaging the eight dendrites selected from each mouse, followed by averaging the mice in each group.^33^ Statistical outliers with values that fell beyond two s.d.'s from the mean were excluded from analysis. Data are reported as mean±s.e.m. Statistical significance was defined at *P*<0.05.

## Results

### Early-life stress reduces DG nectin-3 levels in adulthood

We first analyzed nectin-3 expression levels in the dorsal DG under basal conditions on P2, P9 and P90 to evaluate the temporal expression profile of nectin-3 in the developing and adult DG (Supplementary Figure S2A). One-way ANOVA revealed a significant effect of developmental stage on nectin-3 expression in GCL (*F*_2, 6_=13.049, *P*=0.007), hilus (*F*_2, 6_=35.654, *P*=0.00047) and the whole dorsal DG (*F*_2, 6_=150.408, *P*=0.000007). *Post hoc* analyses showed that nectin-3 levels in DG, especially in GCL and hilus, peaked on P9 (GCL: P9 versus P2, *P*<0.05, P9 versus P90, *P*<0.01; hilus: P9 versus P2, *P*<0.001, P9 versus P90, *P*<0.01; total: P9 versus P2, *P*<0.001, P9 versus P90, *P*<0.001; Tukey’s test). We then examined adult mice with or without postnatal stressful experiences (Supplementary Figure S2B), and found that stress significantly reduced DG nectin-3 levels (ML, *t*_14_=2.564, *P*=0.023; hilus, *t*_14_=3.316, *P*=0.005; total, *t*_14_=2.42, *P*=0.03; independent *t*-test). This indicates that early-life stress may lastingly reduce nectin-3 levels in DG neurons.

### Nectin-3 knockdown in both old and young DG neurons mildly increases anxiety and impairs hippocampus-dependent memory

**Figure 1:**
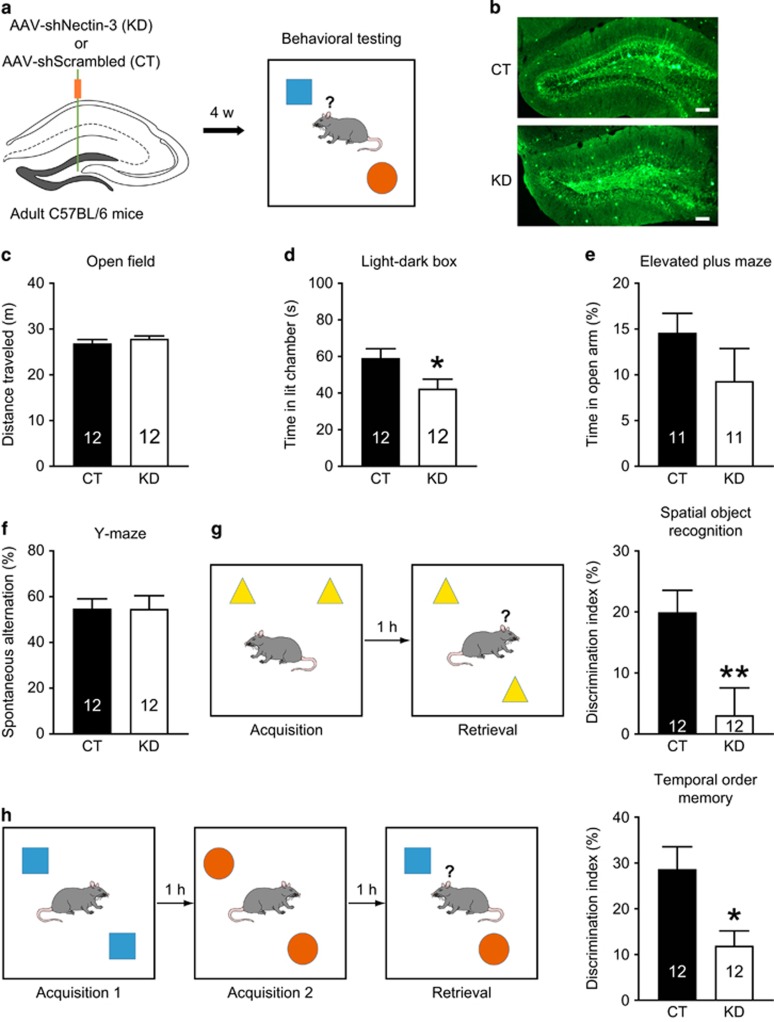
Effects of nectin-3 knockdown in all DG neuron populations on anxiety-related behavior and cognition. (**a**) Schematic of experimental design. (**b**) Representative images showing the expression of enhanced GFP in DG, indicative of neurons infected by AAV-shSCR (the control virus) or AAV-shNEC (the nectin-3 knockdown virus). Scale bars=100 μm. (**c**–**e**) DG nectin-3 knockdown did not affect performance in the open field and elevated plus maze tests, but reduced the time spent exploring the brightly illuminated compartment in the light–dark box test. (**f**) Both groups showed similar spontaneous alternation rates. (**g**, **h**) DG nectin-3 knockdown impaired spatial object recognition memory and temporal order memory. *P<0.05, **P<0.01. In this and subsequent figures, the number of mice is indicated in the bar graphs. AAV, adeno-associated virus; CT, control; DG, dentate gyrus; GFP, green fluorescent protein; KD, nectin-3 knockdown.

To examine the consequences of dorsal DG nectin-3 knockdown on anxiety and cognition in adult mice, we used the AAV-shNEC virus to suppress nectin-3 expression in both old and young DG neurons (Figures 1a and b). AAV-shNEC significantly reduced nectin-3 protein levels in the dorsal DG but not in the CA1–3 regions (DG, *t*_10_=3.193, *P*=0.0096; CA1–3, *t*_10_=−0.003, *P*=0.998; independent *t*-test; Supplementary Figures S3A and B). In addition, DG nectin-3 protein levels were comparable between mice injected with the control virus (AAV-shSCR) and mice without viral injection (Supplementary Figure S3C). For mice that underwent behavioral testing, the suppression of DG nectin-3 protein levels was validated (ML, *t*_21_=3.563, *P*=0.002; hilus, *t*_21_=2.745, *P*=0.012; total, *t*_21_=2.872, *P*=0.009; independent *t*-test; Supplementary Figure S3D). Compared with mice injected with AAV-shSCR, those with decreased DG nectin-3 levels exhibited comparable exploration time in the center zone (control (CT): 47.82±7.5 s, knockdown (KD): 71.36±16.4 s; *t*_15.41_=1.306, *P*=0.211; independent *t-*test) and total distance traveled (Figure 1c) in the open field arena, but spent less time to explore the anxiogenic and brightly lit compartment (*t*_22_=2.125, *P*=0.045; independent *t-*test; Figure 1d). In the elevated plus maze test, no difference was found between groups (Figure 1e). In the Y-maze spontaneous alternation task, which evaluates spatial working memory and depends on multiple brain regions including the hippocampus,^34^ the two groups performed similarly, indicating that DG nectin-3 knockdown did not alter short-term spatial memory (Figure 1f). However, in the hippocampus-dependent spatial object recognition test, nectin-3 knockdown mice showed impaired object discrimination, indicative of long-term spatial memory deficits (*t*_22_=2.853, *P*=0.009; independent *t*-test; Figure 1g). Moreover, nectin-3 knockdown mice had impaired temporal order memory that involves the hippocampus and several neocortical regions^35, 36^ (*t*_22_=2.744, *P*=0.012; independent *t*-test; Figure 1h). In addition, in the acquisition phase(s) of the spatial object recognition and temporal order memory tests, both groups of mice performed similarly (Supplementary Figure S4). Taken together, these results suggest that nectin-3 knockdown in the dorsal DG specifically impairs long-term hippocampus-dependent memory and mildly increases anxiety.

### Nectin-3 knockdown hampers the differentiation and maturation of adult-born DG granule cells

**Figure 2:**
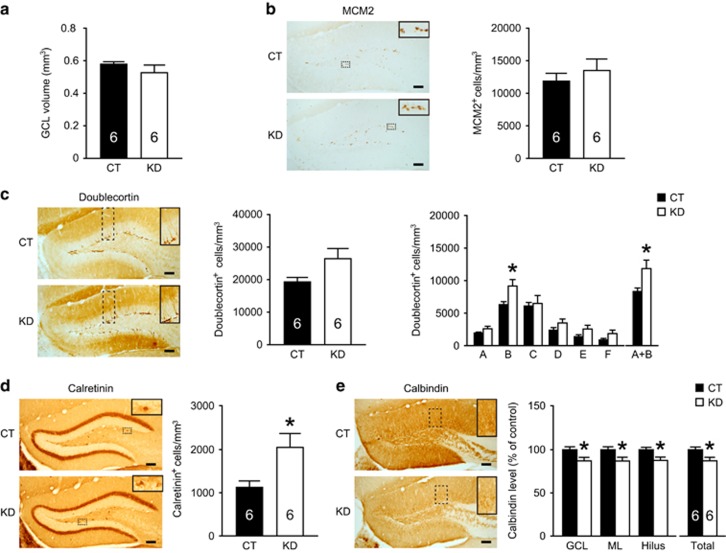
Effects of nectin-3 knockdown on the proliferation, differentiation and maturation of adult-born DG granule cells. (**a**) The volume of dorsal DG GCL was comparable between groups. (**b**) Nectin-3 knockdown did not alter the density of cells immunoreactive for MCM2, a marker of cell proliferation. Inserts show magnified images of MCM2-positive proliferating cells. (**c**) Nectin-3 knockdown did not significantly change the average density of cells immunoreactive for doublecortin, a marker of cell differentiation, but increased the number of category B and categories A+B doublecortin-positive cells. Insets show magnified images of doublecortin-positive cells of different morphology. (**d**) Nectin-3 knockdown increased the density of immature granule cells immunoreactive for calretinin. Insets show magnified images of calretinin-positive immature DG neurons. (**e**) Nectin-3 knockdown significantly reduced the levels of calbindin, a marker of mature granule cells, in all DG subregions. Insets show magnified images of calbindin-positive mature DG neurons. All scale bars=100 μm. *P<0.05. CT, control; DG, dentate gyrus; GCL, granule cell layer; KD, nectin-3 knockdown; MCM2, minichromosome maintenance complex component 2.

We have reported that nectin-3 knockdown in the adult hippocampus reduces dendritic spine density in CA3, CA1 and DG principal neurons.^21^ It is therefore conceivable that nectin-3 is important for spine remodeling in mature hippocampal neurons. However, as AAV also infects dividing cells and non-dividing immature neurons,^37^ it is possible that nectin-3 knockdown disrupts the development and maturation of adult-born dentate granule cells, which may contribute to cognitive deficits. To test this possibility, we used various markers to label DG neurons undergoing proliferation (MCM2), differentiation (doublecortin) or maturation (calretinin and calbindin).^38, 39^ The volume of dorsal GCL was comparable between groups (Figure 2a). Nectin-3 knockdown did not affect cell proliferation as indicated by unaltered density of MCM2-immunoreactive cells in the adult DG (Figure 2b). In nectin-3 knockdown mice, although the average density of doublecortin-immunoreactive cells remained relatively unchanged (*t*_6.806_=2.116, *P*=0.073; independent *t-*test), the number of category B and categories A+B cells significantly increased (category B, *t*_10_=2.606, *P*=0.026; categories A+B, *t*_10_=2.476, *P*=0.033; independent *t*-test; Figure 2c), indicating that nectin-3 may modulate neuronal differentiation and initial dendritic growth. Moreover, nectin-3 knockdown increased the number of calretinin-expressing immature DG neurons in the subgranular zone (*t*_7.009_=2.618, *P*=0.034; independent *t-*test; Figure 2d), but markedly reduced calbindin levels in all DG subregions (GCL, *t*_10_=2.45, *P*=0.034; ML, *t*_10_=2.356, *P*=0.04; hilus, *t*_10_=2.585, *P*=0.027; total, *t*_10_=2.483, *P*=0.032; independent *t*-test; Figure 2e). These data suggest that impaired nectin-3-mediated cell adhesion disrupts the differentiation and maturation of adult-born DG neurons.

### Nectin-3 knockdown in adult-born DG granule cells impairs memory and reduces spine density

**Figure 3:**
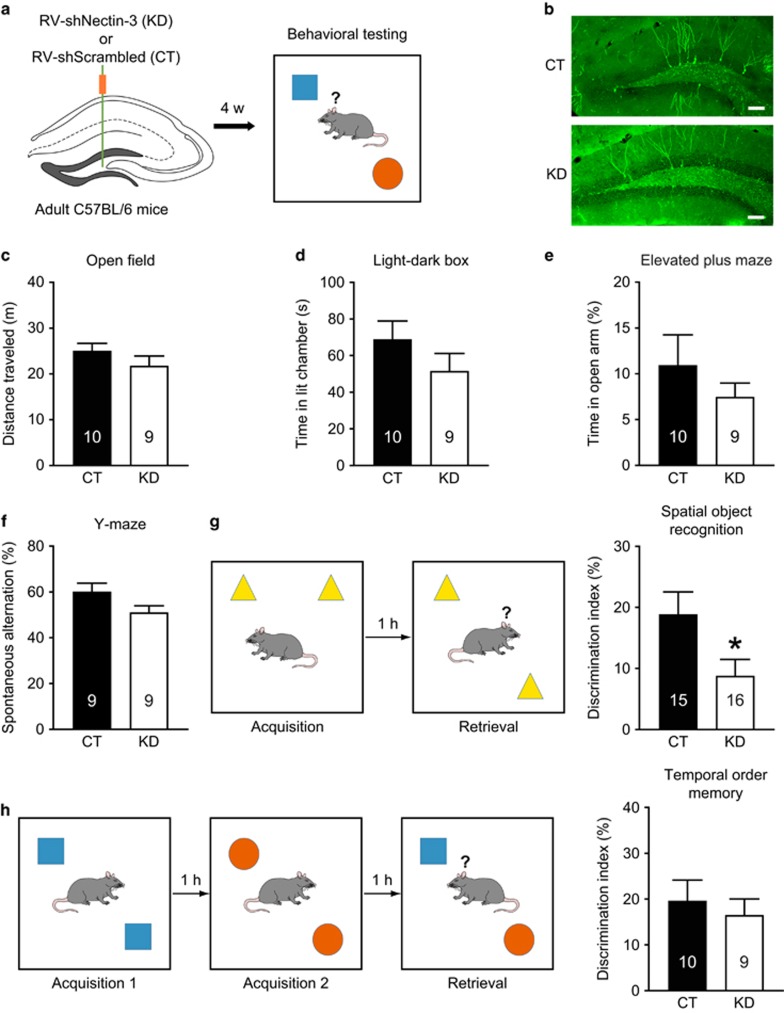
Effects of selective nectin-3 knockdown in adult-born dentate granule cells on anxiety-related behavior and cognition. (**a**) Schematic of experimental design. (**b**) Representative images showing the expression of enhanced GFP in DG, indicative of neurons infected by RV-shSCR (the control virus) or RV-shNEC (the nectin-3 knockdown virus). Scale bars=100 μm. (**c**–**f**) Nectin-3 knockdown in newly generated DG neurons had no effect on anxiety-related behavior and short-term spatial working memory. (**g**, **h**) Nectin-3 knockdown in new DG neurons impaired long-term spatial memory but not temporal order memory. *P<0.05. CT, control; DG, dentate gyrus; GFP, green fluorescent protein; KD, nectin-3 knockdown; RV, retrovirus.

As new DG granule cells are continuously generated in the adult hippocampus, we used RV to examine whether specific knockdown of nectin-3 in young DG neurons disrupts hippocampus-dependent memory (Figures 3a and b). The knockdown efficiency of RV-shNEC was validated as shown by significantly reduced hippocampal nectin-3 protein levels in adult mice with intrahippocampal RV-shNEC injection on P2 (*t*_8_=4.501, *P*=0.002; independent *t-*test; Supplementary Figure S5). In the open field test, no difference in the time spent exploring the center zone (CT: 19.69±4.16 s, KD: 19.62±5.15 s; *t*_17_=0.01, *P*=0.992; independent *t-*test) or total distance traveled was found (Figure 3c). In the light–dark box and elevated plus maze tests, the two groups of mice also performed similarly, indicating that reducing nectin-3 levels in newly generated DG neurons do not alter anxiety (Figures 3d and e). Short-term spatial working memory was not affected by nectin-3 knockdown in new DG neurons (Figure 3f). However, in the spatial object recognition test, mice with nectin-3 downregulated in new DG neurons showed memory impairments as indicated by significantly reduced object DI (*t*_29_=2.157, *P*=0.039; independent *t-*test; Figure 3g). By contrast, in the temporal order memory test, mice with nectin-3 knockdown in new DG neurons had intact memory (Figure 3h). In addition, both groups of mice explored the two presented objects similarly in the acquisition phase(s) of the spatial object recognition and temporal order memory tests (Supplementary Figure S6).

**Figure 4:**
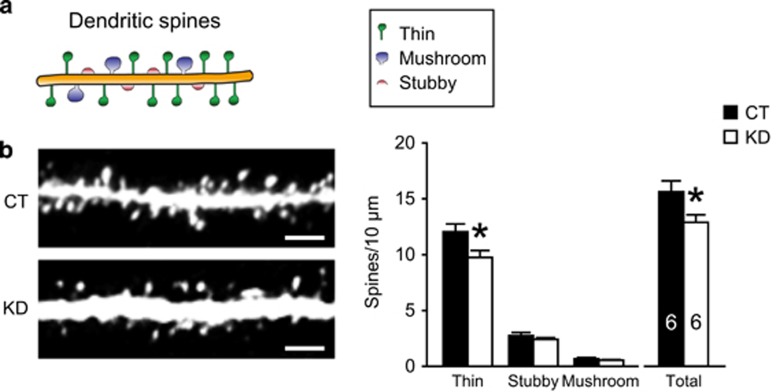
Effects of nectin-3 knockdown in adult-born neurons on spine density in the medial ML of DG. (**a**) Dendritic spines were categorized as thin (long and thin protrusions with a bulbous head), mushroom (protrusions with a small neck and a large head) and stubby (protrusions closely connect to the dendritic shaft and lack a clear neck) subtypes. (**b**) Nectin-3 knockdown in young granule cells reduced the number of spines, especially thin spines, in the dentate medial ML. For each mouse, eight dendritic segments were analyzed. *P<0.05. DG, dentate gyrus; ML, molecular layer.

We further examined the impact of nectin-3 downregulation on spine density in new DG granule cells (Figure 4), and observed that nectin-3 knockdown evoked a reduction of spines, especially thin spines, in the medial ML (thin: *t*_10_=2.441, *P*=0.035; total: *t*_10_=2.318, *P*=0.043; independent *t*-test). In comparison, consistent with our previous findings,^19^ spine density in the medial ML of DG remained unchanged in adult mice with early-life stress exposure (Supplementary Figure S7).

## Discussion

The cell adhesion molecule nectin-3 is implicated in chronic stress-induced synaptic abnormalities and cognitive deficits.^21, 24, 25^ In this study, we showed that nectin-3 expression levels in DG could be reduced in adult mice with neonatal stressful experience. Most importantly, DG nectin-3 knockdown in either all neuron populations or newly generated granule cells impaired hippocampus-dependent memory. Furthermore, weakened nectin-3-mediated cell adhesion disrupted the maturation and structural plasticity of adult-born DG neurons. These results provide further evidence on the role of nectin-3 in structural plasticity, memory and stress-related disorders.

During the early postnatal period, nectin-3 is observed in both synaptic sites and adherens junctions in CA3 neurons; however, it selectively localizes at adherens junctions from P14 onwards.^40^ Nectin-3 is essential for the establishment of axon–dendrite contacts,^41^ and conventional knockout of nectin-3 reduces the number of adherens junctions and induces abnormal axonal targeting of DG granule cells.^42^ Here we noticed that the protein expression of nectin-3 in DG was developmentally regulated, and DG nectin-3 levels peaked on P9 when the neonatal hippocampus and neocortex are highly sensitive to severe stress challenge.^4, 28^ Although further evidence on the subcellular localization of nectin-3 in immature and mature DG neurons is needed, we found that nectin-3 levels were significantly reduced by early-life stress in the adult DG, indicative of its potential role in the development of DG neurons.

Elevated hippocampal CRH–CRHR1 signaling has been hypothesized to mediate the effects of early-life stress on cognition. Recently, we have reported that CRH–CRHR1 signaling impairs hippocampus-dependent memory through reducing nectin-3 protein levels in the CA3 region,^21^ indicative of nectin-3 as a downstream molecule of CRHR1. In the present study, we further showed that the suppression of nectin-3 in both immature and mature DG neurons impaired spatial object recognition memory that is specifically dependent on the hippocampus, and temporal order memory that depends on the neuronal networks among the hippocampus, medial prefrontal cortex and perirhinal cortex.^35, 36^ These results indicate that nectin-3 in the DG region may also be a target of early-life stress and CRHR1 signaling. In addition, consistent with our previous findings,^21^ spatial working memory as evaluated by the Y-maze spontaneous alternation task was not altered by DG nectin-3 knockdown. This suggests nectin-3 as a molecular substrate for long-term instead of short-term memory.

A main feature of DG is the continuous generation of new granule cells from subgranular zone throughout life. Adult neurogenesis in DG has been shown to modulate memory and anxiety-related behavior.^38, 39^ A large body of evidence demonstrates that early-life stress reprograms DG neurogenesis, with stress effects varying depending on species, sex, age, genetic background and stress paradigms.^6, 10, 16, 17, 18^ Neonatal and adult neurogenesis in the early-life stress model we used has been intensively characterized by a recent study.^12^ Such postnatal manipulation transiently increases neuronal proliferation and differentiation in the neonatal DG, but lastingly inhibits the survival of neonatal- and adult-born DG neurons. Our data revealed that DG nectin-3 knockdown did not affect cell proliferation, but increased the density of differentiating cells as shown by doublecortin-immunoreactive categories A and B cells. In addition, the density of differentiated and immature DG neurons expressing calretinin was increased, whereas the immunoreactivity of calbindin, which marks mature DG granule cells that have integrated into neuronal circuits, was decreased in nectin-3 knockdown mice. Collectively, these results suggest that nectin-3 contributes to the differentiation and maturation of adult-born DG neurons, and its reduction may partially contribute to early postnatal stress-induced effects on neurogenesis. Moreover, in line with our previous results that hippocampal nectin-3 knockdown reduces spine density in the outer ML of DG,^21^ we found that nectin-3 knockdown specifically in newly generated neurons also reduced the density of spines, especially thin spines in the medial ML, highlighting the importance of nectin-3 in dendritic spine plasticity. Taken together, these data suggest that nectin-3 modulates the development of young DG neurons and remodels the structure of old DG neurons. Reduced nectin-3 levels impair the structural integrity of DG neurons, which in turn contributes to early-life stress-induced memory loss. In future studies, more detailed analyses are needed to dissect the involvement of nectin-3 in different stages of developmental and adult neurogenesis as well as dendritic growth and spine formation/elimination, so that a better understanding on nectin-3-mediated cell adhesion in the structural plasticity of DG neurons could be obtained.

It should be noted that, on the one hand, although nectin-3 knockdown in either all DG neurons or adult-born neurons alone mimicked early-life stress-induced long-term spatial memory deficits, it did not reproduce every aspect of the stress effects. On the other hand, nectin-3 knockdown resulted in molecular and morphological phenotypes that were not prominent in neonatally stressed adult mice. For instance, as shown in this study and previously,^19, 21^ spine density in the ML of DG was altered in nectin-3 knockdown but not in postnatally stressed mice. This may be ascribed to the constellations of molecular and cellular changes evoked by early-life stress,^43, 44^ including neurexins,^19^ neuroligins^45^ and other cell adhesion molecules that may exert compensatory effects. Alternatively, as adherens junctions are present in 33% of dendritic spines in CA1 pyramidal neurons,^46^ nectin-3 knockdown-induced effects may be only responsible for the reduction of a small fraction of spines.

We observed a mild anxiogenic effect of nectin-3 knockdown in all DG neurons. Considering the limitations in the microinjection procedure, this phenotype may be explained by the spreading of AAV-shNEC to part of the ventral DG that modulates anxiety.^8, 9^ In addition, mice injected with AAV-shNEC exhibited anxiety-related behavior only in the light–dark box test but not in the open field or elevated plus maze test. This may be ascribed to the sensitivity of different tests and/or the intra-individual variations in emotionality.^47^ In future studies, it would be interesting to investigate whether specific nectin-3 knockdown in ventral DG neurons indeed increases anxiety.

In summary, our data indicate that nectin-3 modulates the development and plasticity of DG granule cells, which in turn contribute to hippocampus-dependent memory. These findings may shed light on the importance of nectin-3-mediated synaptic and cell adhesion in neuronal development and plasticity, learning and memory, and stress-related psychiatric disorders.
