# Supplementary material for: Nectin-3 modulates the structural plasticity of dentate granule cells and long-term memory
Source: Transl Psychiatry. 2017 Sep 5;7(9):e1228–. doi: 10.1038/tp.2017.196 (PMC5639241; doi:10.1038/tp.2017.196)
Supplement: Supplementary Information [file tp2017196x1.docx]

**Supplementary Information for:**

**Nectin-3 Modulates the Structural Plasticity of Dentate Granule Cells and Long-Term Memory**

Xing-Xing Wang, Ji-Tao Li, Xiao-Meng Xie, Yan Gu, Tian-Mei Si, Mathias V. Schmidt, and Xiao-Dong Wang

**Supplementary Figures**


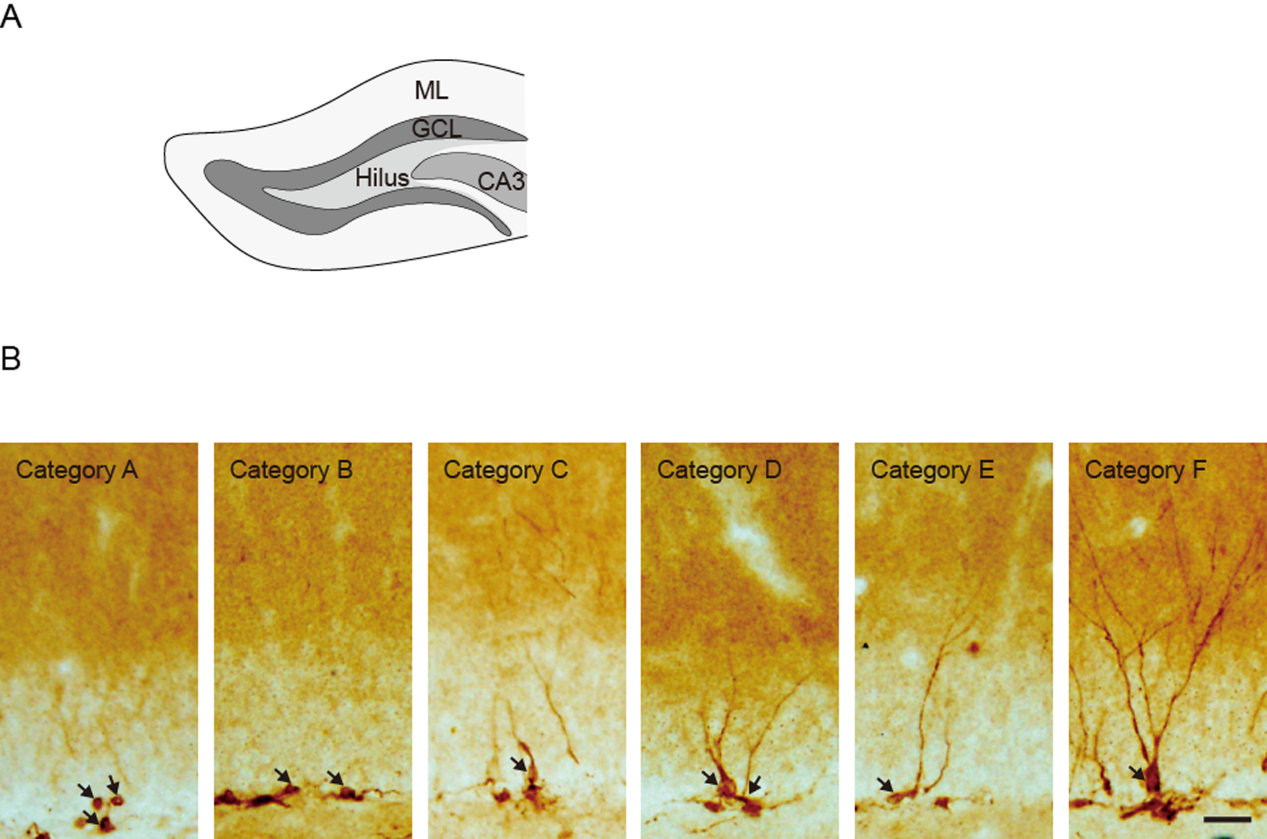
­­­

**Figure S1.** Schematic of the laminated structure of dorsal DG and the categorization of doublecortin-immunoreactive cells. (A) Schematic of the dorsal DG showing the regions of interest. GCL, granule cell layer; ML, molecular layer. (B) Doublecortin-immunoreactive cells in the dentate gyrus are categorized based on dendritic morphology. Category A cells do not have process, and category B cells have short and plump processes, both of which mark cells in the proliferative stage. Category C cells have medium process, while category D cells have process reaching to the molecular layer, both marking cells in the intermediate stage. Category E cells have one primary dendrite branching in the molecular layer, and category F cells have delicate dendritic trees branching in the granule cell layer and molecular layer, both marking cells in the postmitotic stage. Arrows point to doublecortin-immunoreactive cells that fit to the criterion of each stage. Scale bar = 25 μm.


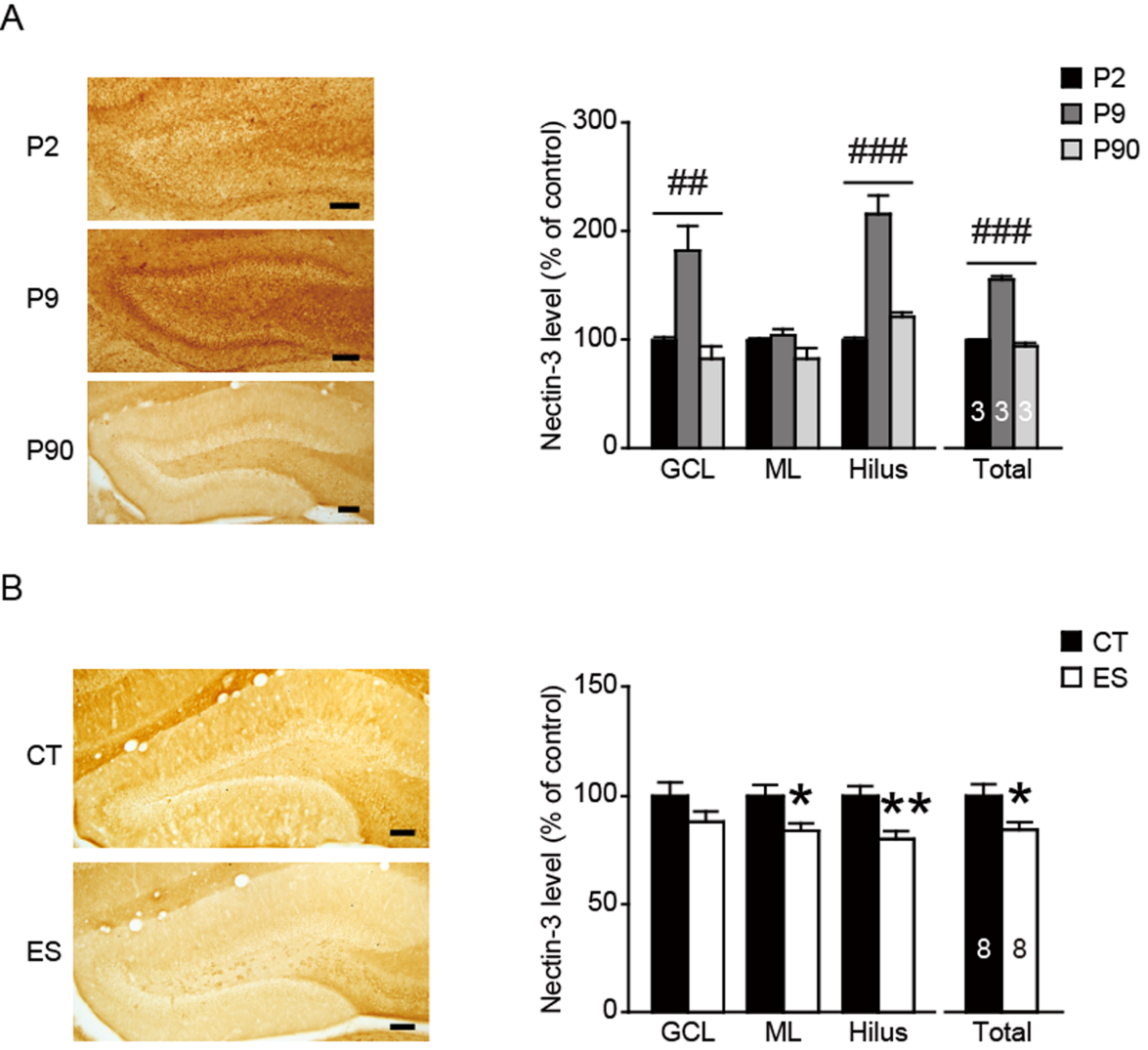


**Figure S2.** Temporal expression pattern of nectin-3 in the dorsal DG and the effects of early-life stress. (A) Nectin-3 immunoreactivity in DG, especially the GCL and hilus, is higher on P9 than on P2 or P90. (B) In neonatally stressed adult mice, nectin-3 levels were reduced especially in the ML and hilus of DG. All scale bars = 100 μm. CT, control; ES, early-life stress. ## *p* < 0.01, ### *p* < 0.001, time effect. * *p* < 0.05, ** *p* < 0.01.


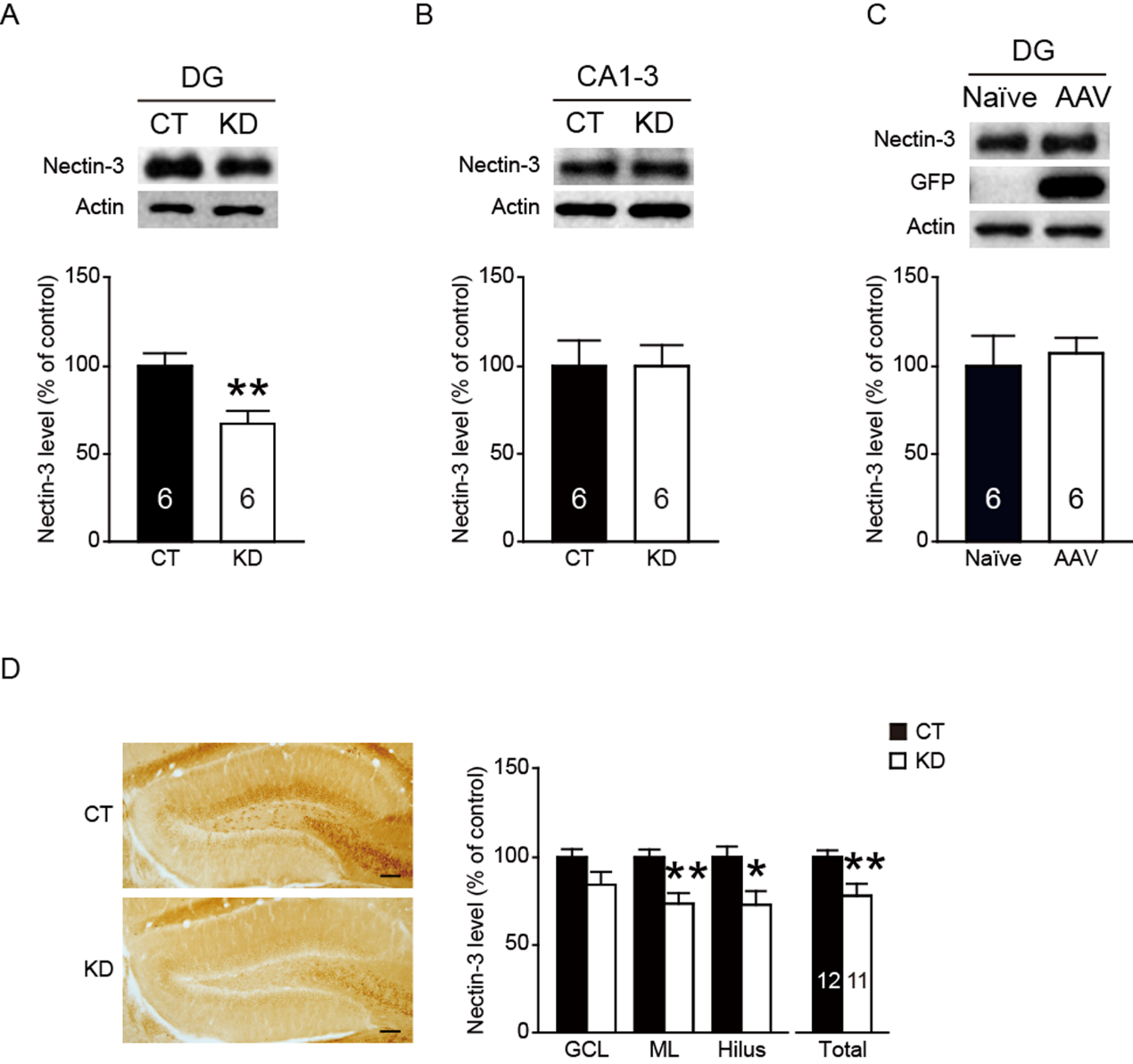


**Figure S3.** Knockdown efficiency and specificity of AAV-shNEC. (A) AAV-shNEC significantly suppressed nectin-3 protein expression in adult DG neurons. (B) Nectin-3 levels in CA1-3 regions were comparable between groups. (C) DG nectin-3 protein levels were comparable bewteen mice injected with AAV-shSCR (AAV) and mice without viral injection (Naïve). (D) For mice used for behaviroal testing, the suppression of DG nectin-3 protein levels was validated. Scale bars = 100 μm. CT, control (AAV-shSCR); KD, knockdown (AAV-shNEC). * *p* < 0.05, ** *p* < 0.01.


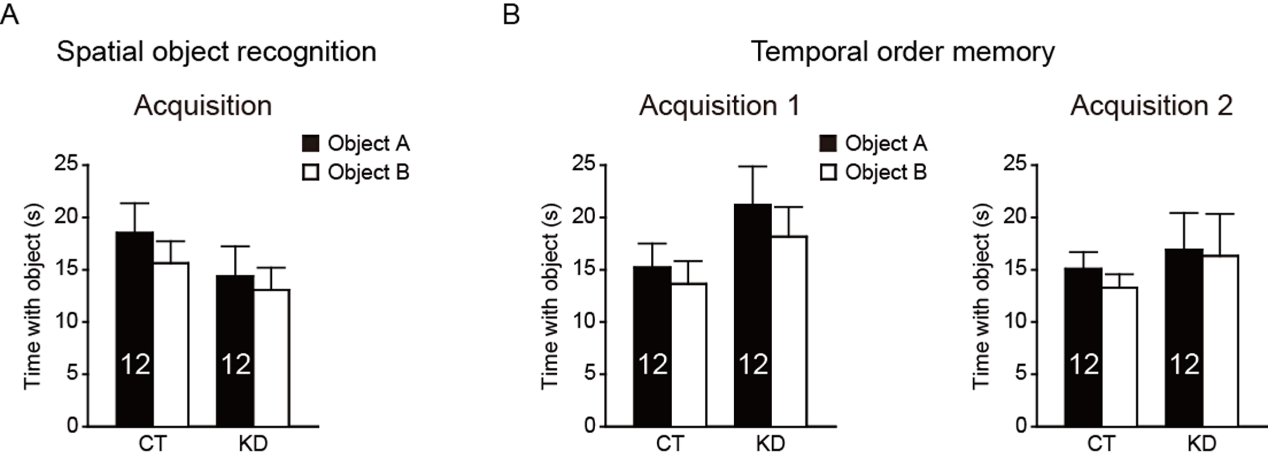


**Figure S4.** (A) In the acquisition phase of the spatial object recognition test, mice with hippocampal microinjection of either AAV-shSCR or AAV-shNEC explored the two presented objects similarly, and no difference between groups was noticed. (B) In the two acquisition phases of the temporal order memory test, both groups of mice explored the presented objects similarly, and no object or location preference was found. CT, control (AAV-shSCR); KD, knockdown (AAV-shNEC).


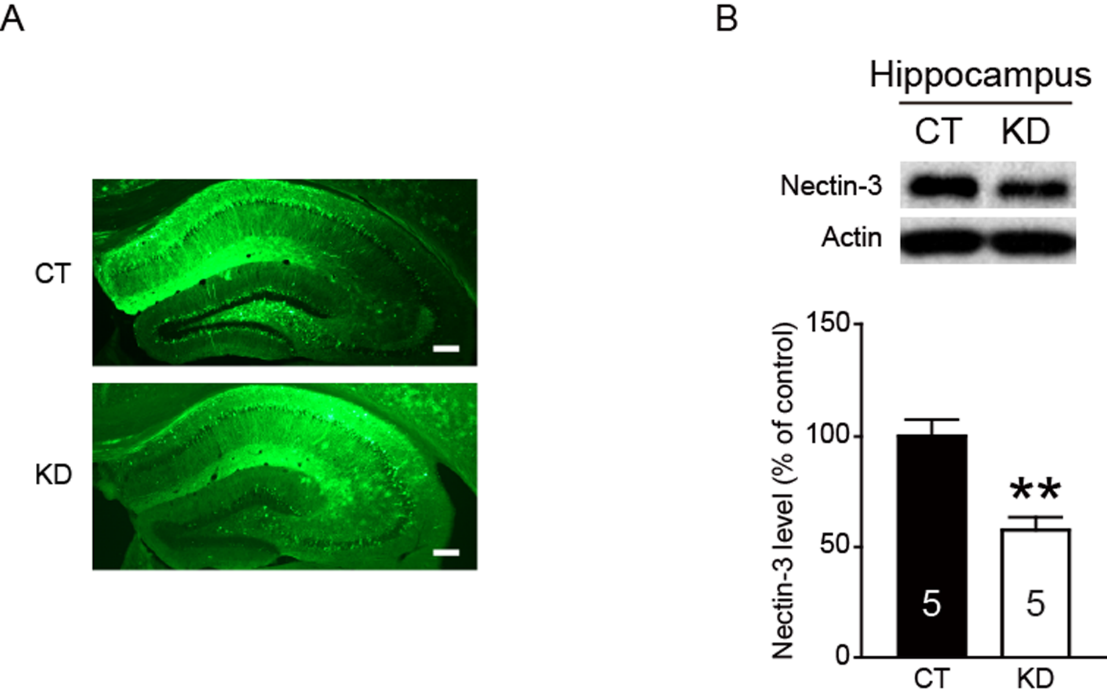


**Figure S5.** Validation of nectin-3 knockdown efficiency of RV-shNEC. (A) Representative immunofluorescent images showing the infection of hippocampal neurons by RV-shSCR or RV-shNEC. The viruses were injected on P2, and mice were killed at 8 weeks of age. Scale bars = 200 um. (B) RV-shNEC significantly reduced hippocampal nectin-3 protein levels in adult mice with intrahippocampal RV-shNEC injection on P2. CT, control (RV-shSCR); KD, knockdown (RV-shNEC). ** *p* < 0.01.


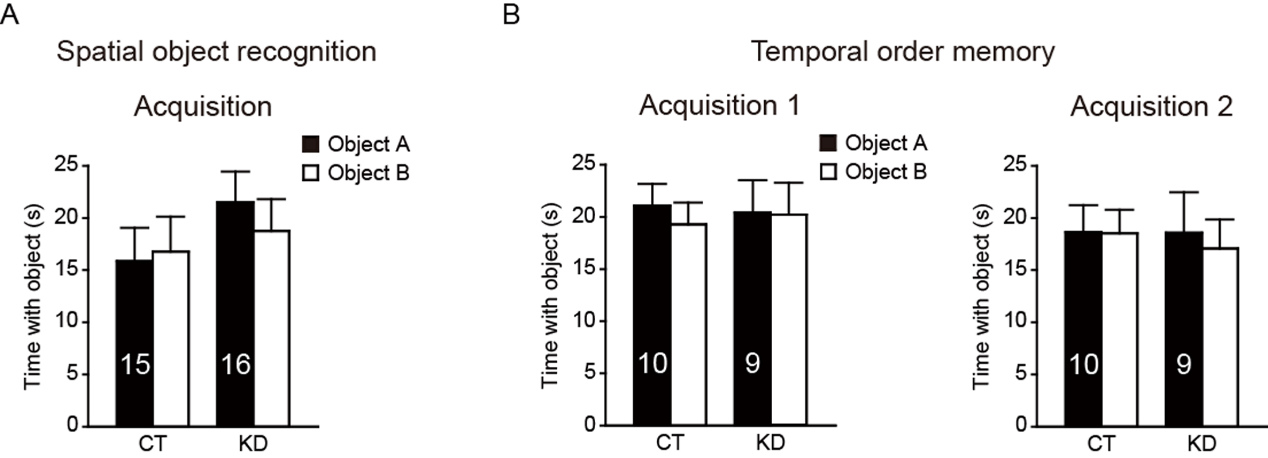


**Figure S6.** (A) In the acquisition phase of the spatial object recognition test, mice with hippocampal microinjection of either RV-shSCR or RV-shNEC explored the two presented objects similarly, and no difference between groups was noticed. (B) In the two acquisition phases of the temporal order memory test, both groups of mice explored the presented objects similarly, and no object or location preference was found. CT, control (RV-shSCR); KD, knockdown (RV-shNEC).


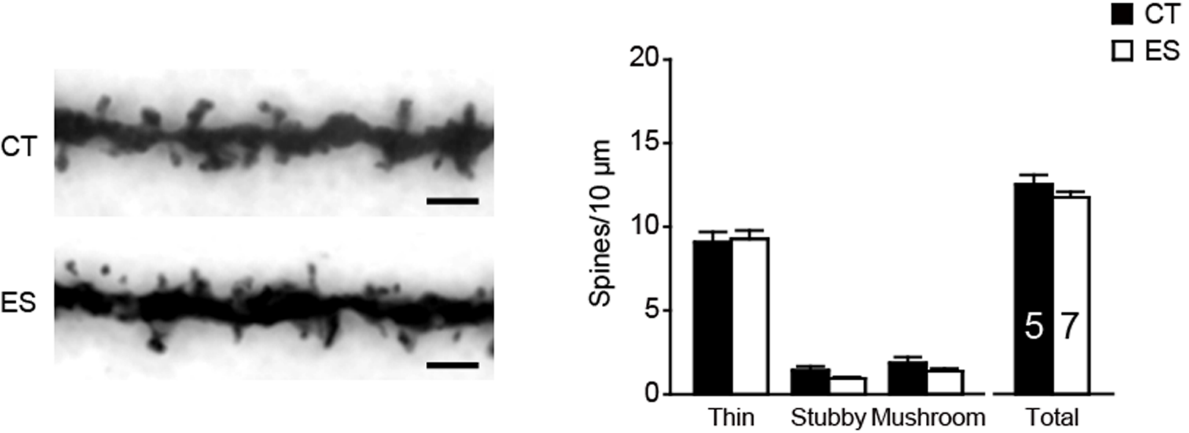


**Figure S7.** In adult mice, early-life stress had no effect on dendritic spine density in the medial molecular layer of DG. Scale bars = 2 μm. CT, control; ES, early-life stress.
